# Toxin Production by *Stachybotrys chartarum* Genotype S on Different Culture Media

**DOI:** 10.3390/jof6030159

**Published:** 2020-09-02

**Authors:** Sebastian Ulrich, Cornelius Schäfer

**Affiliations:** 1 Chair of Bacteriology and Mycology, Faculty of Veterinary Medicine, Ludwig-Maximilians-University Munich, Veterinärstraße 13, 80539 Munich, Germany; 2 BÜCHI Labortechnik GmbH, Altendorfer Straße 3, 45127 Essen, Germany; c.schaefer@buchi.com

**Keywords:** *Stachybotrys*, genotype, macrocyclic trichothecenes, stachybotrylactam

## Abstract

*Stachybotrys* (*S*.) *chartarum* had been linked to severe health problems in humans and animals, which occur after exposure to the toxic secondary metabolites of this mold. *S. chartarum* had been isolated from different environmental sources, ranging from culinary herbs and improperly stored fodder to damp building materials. To access the pathogenic potential of isolates, it is essential to analyze them under defined conditions that allow for the production of their toxic metabolites. All *Stachybotrys* species are assumed to produce the immunosuppressive phenylspirodrimanes, but the highly cytotoxic macrocyclic trichothecenes are exclusively generated by the genotype S of *S. chartarum*. In this study, we have analyzed four genotype S strains initially isolated from three different habitats. We grew them on five commonly used media (malt-extract-agar, glucose-yeast-peptone-agar, potato-dextrose-agar, cellulose-agar, Sabouraud-dextrose-agar) to identify conditions that promote mycotoxin production. Using LC-MS/MS, we have quantified stachybotrylactam and all S-type specific macrocyclic trichothecenes (satratoxin G, H, F, roridin E, L-2, verrucarin J). All five media supported a comparable fungal growth and sporulation at 25 °C in the dark. The highest concentrations of macrocyclic trichothecenes were detected on potato-dextrose-agar or cellulose-agar. Malt-extract-agar let to an intermediate and glucose-yeast-peptone-agar and Sabouraud-dextrose-agar to a poor mycotoxin production. These data demonstrate that the mycotoxin production clearly depends on the composition of the respective medium. Our findings provide a starting point for further studies in order to identify individual components that either support or repress the production of mycotoxins in *S. chartarum*.

## 1. Introduction

*Stachybotrys (S.) chartarum* is the most frequently isolated species of the genus *Stachybotrys* [1,2] and had been isolated from dead plant materials (e.g., herbs, straw, and hay) and other cellulosic, and water damaged substrates (e.g., wallpaper, plasterboard, or wooden lining) [3,4,5,6]. The species *S. chartarum* can be further subdivided into three genotypes, the highly cytotoxic genotype S that produces macrocyclic trichothecenes (MCT), the low cytotoxic genotype A that produces atranone and the recently described genotype H that produces phenylspirodrimanes, but no macrocyclic trichothecenes [7].

*S. chartarum* S-type strains produce satratoxins and other MCT [8]. By binding irreversibly to the 60S ribosomal subunit, satratoxins inhibit protein biosynthesis [9,10,11] and represent the most cytotoxic trichothecenes currently known [12]. The genotype S had been implicated in several types of disease, such as stachybotryotoxicosis in animals, hemorrhage in human infants, and the sick building syndrome [13,14,15,16,17,18,19,20,21,22,23] Stachybotryotoxicosis may occur after oral uptake in horses as well as in cattle and sheep [19,20,24]. In humans, exposure to macrocyclic trichothecenes (MT) may cause pulmonary hemorrhage in infants or symptoms related to the sick building syndrome complex, mainly due to fungal growth on building materials after water damages [21,22,23].

Therefore, it is surprising how little is known about the influence of nutritional factors on their production. This point is particularly important since reliable protocols are needed to determine individual strains’ ability to produce mycotoxins and thereby evaluate their harmful potential. To address this, we have designed a study in which four genotype S strains were grown on five different media that are used on a routine basis for fungi identification [25]. Research on mycotoxin production on various building materials was done for MCT and phenylspirodrimanes [18,26,27]. Building materials and natural habitats of *S. chartarum* genotype S are too complex media to identify limiting nutritional factors. Factors such as temperature and humidity are already known to influence the MCT production [6,28,29]. Thus, this study is a comparison of different media under standardized conditions regarding temperature and humidity. To our best knowledge, no study has so far analyzed the influence of different in vitro media on the MCT production.

Two field isolates and two reference strains were selected for this study. These isolates originated from three different habitats (building material, animal feed, and foodstuff). They were cultivated as three-point cultures on five different culture media (MEA: malt-extract-agar, GYP: glucose-yeast-peptone-agar, PDA: potato-dextrose-agar, CEL: cellulose-agar, SAB: Sabouraud-dextrose-agar) with three biological replications for 21 days in the dark. The MCT (satratoxin G, H, F, roridin E, roridin L-2, verrucarin J) and stachybotrylactam were analyzed by LC-MS/MS. For this purpose, we developed and validated an inhouse LC-MS/MS method. Using this approach, we have identified media that support or prevent the production of the analyzed mycotoxins.

## 2. Materials and Methods

### 2.1. Chemicals

LC-MS-grade acetonitrile (ACN), methanol, formic acid, and ammonium formate used as additives for LC-MS solvents were purchased from Th. Geyer (Renningen, Germany) and Fluka (Steinheim, Germany), respectively. Ultrapure water was obtained by purifying water through an UltraClear^®^ TP UV UF TM from Evoqua Water Technologies (Barsbuettel, Germany). Standards for roridin E (RE) and L-2 (RL-2), satratoxins F (SF), G (SG), and H (SH) and verrucarin J (VJ) were not commercially available, and these toxins were therefore qualitatively determined after the purification of these substances from rice cultures as described earlier [30]. Roridin A (RA) and verrucarin A (VA) were purchased from Markor Chemicals Ltd. (Shanghai, China), and the MCT were quantified as RA analog [31]. Stachybotrylactam (Stl) was purchased from Sigma-Aldrich (Merck KGaA, Darmstadt, Germany).

### 2.2. Fungal Cultures and Culture Conditions

In this study, four *S. chartarum* genotype S strains were used, comprising two field (SB01a; S16St.) and two reference strains (IBT40293; ATCC34916)(available at ATCC (American Type Culture Collection), IBT Culture Collection of Fungi, Denmark and Culture Collection of the Chair of Food Safety, Faculty of Veterinary Medicine, Ludwig-Maximilians-University Munich). SB01a was isolated from oregano [3], S16St. from straw, IBT40293 from building materials [32] and ATCC34916 from oats. These strains were initially characterized using previously described molecular and mass spectrometric methods [7,32,33] and were confirmed as *S. chartarum* genotype S. For long-term preservation, fungal cultures were treated as described by Niessen and Vogel [34] and maintained at −80 °C. Working cultures of *Stachybotrys* spp. were grown on 2% malt extract agar plates (MEA, per liter 20 g malt extract, 2 g soy peptone, and 15 g agar (Merck, Darmstadt, Germany), adjusted to pH 5.4). Before use, all media (MEA: malt-extract-agar, GYP: glucose-yeast-peptone-agar, PDA: potato-dextrose-agar, CEL: cellulose-agar, SAB: Sabouraud-dextrose-agar; Ingredients: Table 1) were sterilized by autoclaving at 121 °C for 15 min. All cultures were grown at 25 °C and 95% rel. air humidity as three-point cultures in the dark (Figure S1).

### 2.3. Statistical Analysis

For statistical analysis, the Software OriginPro 2020 (64-bit) SR1 (Version 9.7.0.188) was used. Distribution of the mycotoxin contents between the different nutrition media was tested for significance applying the Kruskal–Wallis ANOVA test and the Mann–Whitney test.

### 2.4. LC-MS/MS Analysis

#### 2.4.1. Extraction Method

For MCT and Stl analysis, each strain was cultured on three parallel MEA, GYP, PDA, CEL, and SAB, as described above. Cultures were stored at −20 °C until extraction. Before extraction, each of the three parallel plates were separately transferred to a mixing bag, and 50 mL ACN/H2O (84/16, *v*/*v*) were added. Bags were treated for 5 min in a bag mixer (BagMixer 400, InterScience, St Nom la Bretèche, France). The sample extracts were filtered through a paper filter, and 10 mL were filtered with an SPE cartridge (Strata-X 33u Polymeric Reversed-Phase 600 mg/6 mL, Phenomenex, Aschaffenburg, Germany). An aliquot of 5 mL was diluted 1:10 and evaporated to dryness under a gentle flow of nitrogen at 50 °C. The residues were resuspended in 1 mL ACN/H_2_O (30/70, *v*/*v*) using ultrasonication (5 min) and were subsequently filtered through a PVDF (polyvinylidene fluoride) syringe filter (0.45 μm, Berrytec, Grünwald, Germany) into a 1.5 mL glass sample vial.

#### 2.4.2. LC-MS/MS Measurement and Method Performance

The LC-MS/MS system consisted of an HPLC apparatus (Shimadzu LC-20AB, SIL-20AC HT, CTO-20AC, CBM-20A, Duisburg, Germany) and an API 4000 triple quadrupole mass spectrometer (Sciex, Darmstadt, Germany). The parameters were previously described in Ulrich et al. [7]. Analyst (Version 1.6.2) and MultiQuant software (Version 3.0.1), both provided by Sciex (Darmstadt, Germany), were used for data acquisition and processing.

Matrix-matched calibration was done separately for every nutrition media tested. Matrix standard solutions were prepared with the obtained extracts. The recovery rate and precision of the method were defined. The previously described toxin standards and S8-extract [31] were used as artificial contaminants to spike the matrix in triplicate (40 ng/g and 100 ng/g). The precision at concentrations of 40 ng/g and 100 ng/g was between 2.9% and 5.3%, respectively, 2.5% and 7.6%. Solvent calibration (5, 10, 25, 50, and 100 ng/g) and matrix calibration were done to define the matrix effect. Especially GYP had a matrix suppression effect of up to 30%, which was the highest compared to the other nutrition media. The recovery rates are summarized in Table S1 and were between 49 and 136% with a relative standard deviation of 0.1–11.3%.

For the limit of detection (LOD) and limit of quantification (LOQ), the signal of noise of 3 and 9 was used, respectively. The LOD and LOQ are shown in Table 2.

## 3. Results and Discussion

**Figure 1:**
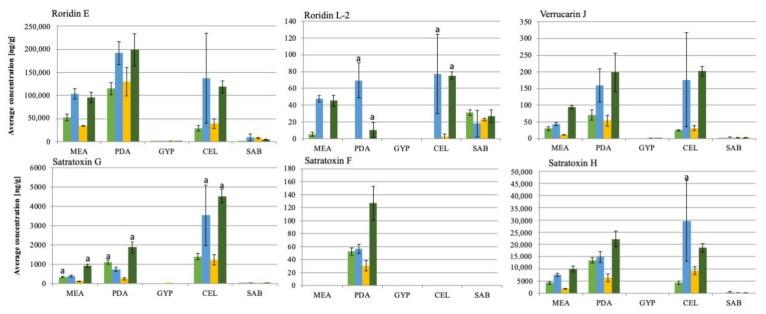
Average mycotoxin concentration (*n* = 3) of six macrocyclic trichothecenes on different nutrition media of four different *Stachybotrys chartarum* genotype S strains (from left to right: S16St.; SB01a; IBT40293; ATCC34916); MEA: Malt-extract-Agar; PDA: Potato-Dextrose-Agar; GYP: Glucose-Yeast-Peptone-Agar; CEL: Cellulose-Agar; SAB: Sabouraud-Glucose-Agar; a: indicates significant differences in concentrations compared to other strains (*p* < 0.05).

The four genotype S strains grew well and showed comparable sporulation levels on all five solid media (Figure S1). The latter is particularly important since mycotoxin production and sporulation are assumed to be linked in *S. chartarum* [35]. The LC/MS analysis of the fungal extracts revealed that the overall pattern of mycotoxins was roughly comparable for the four strains, but IBT40293 and S16St. produced lower concentrations of mycotoxins than the two other strains, indicating that strain-specific differences exist, but primarily with respect to concentrations of the mycotoxins. The MCT concentrations detected for the individual strains on different nutrition media are shown in Figure 1.

**Figure 2:**
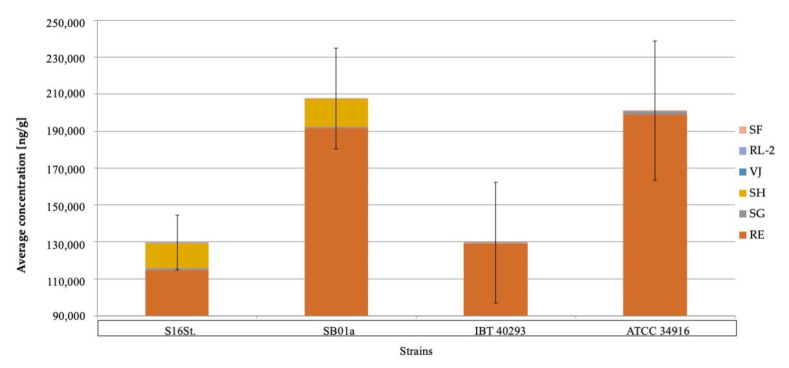
Shows the total amount of macrocyclic trichothecenes (MCT) detected on PDA for every strain since this was the nutrition medium with the highest amounts of MCT. In PDA, strains S16St. and IBT40293 produced significantly (*p* < 0.05) fewer toxins than the other two strains. The total concentration of macrocyclic trichothecenes of four different *Stachybotrys chartarum* genotype S strains on potato-dextrose-agar (PDA). RE: roridin E; RL-2: roridin L-2; SF: satratoxin F; SG: satratoxin G; SH; Satratoxin H; VJ: verrucarin J; Stl.: stachybotrylactam.

The concentrations of the individual mycotoxins differed substantially (Figure 2). The highest concentrations were measured for roridin E (198,956.6 ng/g), satratoxin H (29,601.4 ng/g) and satratoxin G (4520.4 ng/g), whereas lower concentrations of verrucarin J (202.4 ng/g), satratoxin F (127.0 ng/g) and roridin L-2 (77.3 ng/g) were found. Verrucarin A was not produced by any of the strains, which is in line with previous studies [36,37].

Growth on PDA and CEL, and to a minor extend also on MEA, favored the mycotoxin production, whereas GYP and SAB had a negative impact except for roridin L-2 that was produced by all strains during growth on SAB. The most apparent difference between PDA and CEL on the one, and GYP and SAB on the other side is that the former media contain polysaccharides. Previously published data showed that animals and humans were affected by toxins that *S. chartarum* produced during growth on cellulose-rich materials, e.g., wallpaper and straw [19,38,39]. Croft et al. [38] assumed that a high content of cellulose and low content of nitrogen stimulate *Stachybotrys* spp. to produce secondary metabolites such as satratoxins. An impact of nitrogen on the production of secondary metabolites is well established for other fungi and is mediated by an array of nitrogen regulation mechanisms [40]. An impact of nitrogen is also supported by the finding that growth on SAB and GYP, which contain 5 and 10 g/L of peptone, respectively, resulted in low levels of mycotoxins, whereas PDA and CEL that lack peptone and contain lower levels of nitrogen favored the production of mycotoxins by *S. chartarum*. Remarkably, all mycotoxins analyzed in this study lack nitrogen [41]. It is conceivable that a good supply with nitrogen favors the synthesis of other secondary metabolites.

Moreover, there is evidence for mutual influence between secondary metabolism and fungal development, in particular sporulation [35]. The regulation of both processes is well known to be controlled by the available C- and N-source. Further experiments are clearly required to determine the impact of nutrients on the production of mycotoxins in *S. chartarum* and the data of the current study provide an excellent starting point for such a study. No, or minor, concentrations of MCT were detectable after growth on GYP and SAB (<LOD to 629.3 ng/g). This finding suggests that glucose and peptone are not supportive for the production of the toxins analyzed.

Interestingly, strains IBT40293 and S16St. always produced almost one third less on all nutrition media than the two strains SB01a and ATCC34916; whether this is because of laboratory adaptation or a consequence of strain-specific traits is unknown.

In the following, the single investigated MCT are discussed, starting with RE, the presumed precursor of all satratoxins [41]. On MEA and CEL amounts of 28,822.6 ± 6084.2 to 136,961.0 ± 97,289 ng/g were detected. Therefore, RE is the most abundant MCT. Aleksic et al. [26] identified SH (51–64% of the total amount of detected MCT) and SG as the most abundant MCT on building materials, but they had not analyzed their samples for RE. In previous studies on naturally invested materials, SH was the most abundant [38,42,43], but, again, RE was not investigated or not quantified. Future studies should investigate whether RE is also the most abundant MCT on building materials.

According to the proposed synthesis pathway by Degenkolb et al. [44] RL-2 is a product derived from RE. All strains produced small amounts of RL-2 on SAB and non on GYP. Only two strains, SB01a and ATCC34916, generated RL-2 on PDA, but these strains produced much higher amounts on MEA, PDA, and CEL. On PDA, these strains generated significantly different levels (*p* < 0.05; Figure 1 RL-2 a) of this toxin; SB01a produced 69.6 ± 20.7 ng/g and ATCC34916 10.7 ± 9.0 ng/g. On CEL and MEA, both strains produced similar amounts of RL-2 (approx. 80 ng/g).

VJ is another putative product of RE and the presumed precursor of Verrucarin B, which is the precursor of SG [44]. VJ was most abundantly produced on PDA and CEL, whereas only small amounts were detectable on MEA and SAB. On GYP, the concentrations for strains S16St. and SB01a were under the LOD. Large amounts were only produced by the strains SB01a and ATCC34916 (159.6 ± 49.8 ng/g and 202.4 ± 14.7 ng/g) on PDA and CEL.

SG is the first satratoxin product derived from Verrucarin B [44]. SG was particularly prominent on CEL for all strains. This finding is in line with previous studies on cellulose-rich materials, e.g., straw and wallpaper [26,45]. For SB01a and ATCC34916 3534.1 ± 1557.2 ng/g and 4520.5 ± 343.6 ng/g of SG were detected on CEL, respectively. There was a significant (*p* < 0.05; Figure 1 SG a) difference in toxin production on MEA, PDA, and CEL for these two strains.

SH was the second most abundantly detected MCT (29,601 ± 16,542.4 ng/g) and produced in high amounts on CEL and PDA. Strain SB01a produced significantly higher (*p* < 0.05; Figure 1 SH a) amounts of SH on CEL (29,601 ± 16,542.4 ng/g) compared to the other strains. Even if the strain also had the highest variation between the biological replicates. The variance of SH production between the strains was only significant (*p* < 0.05) on CEL. Aleksic et al. (2016) [26] detected SH as the primary MCT on wallpaper, with concentrations of 14.2 mg/m^2^.

SF is another satratoxin differing from SG only in one missing hydroxy-group [46]. SF was only detectable in minor amounts and only on PDA. The lowest amount of this toxin was found for strain IBT40293 (30.5 ± 7.7 ng/g SF). Strains S16St. and SB01a produced intermediate concentrations of approximately 60 ng/g SF and strain ATCC34916 produced the highest concentration (127.0 ± 25.98 ng/g). Nevertheless, the comparably low concentrations of SF could be explained by the suggestion of Degenkolb et al. [44] that SF is the last product in the MCT synthesis and thus proportionally lower in its concentration than other MCT.

**Figure 3:**
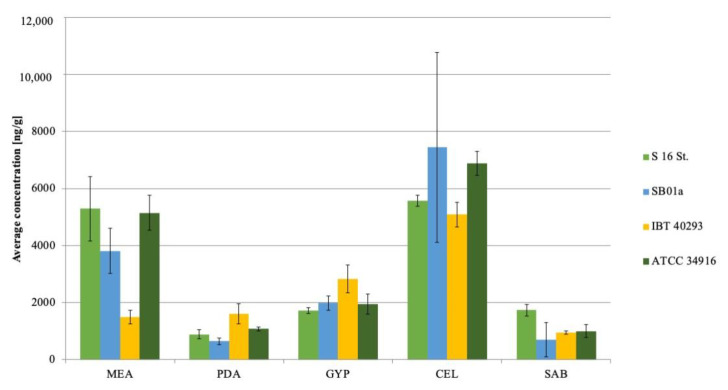
Median concentration (*n* = 3) of stachybotrylactam of four different *Stachybotrys chartarum* genotype S strains on five different nutrition media. MEA: Malt-extract-agar; PDA: Potato-dextrose-agar; GYP: Glucose-yeast-peptone-agar; CEL: Cellulose-agar; SAB: Sabouraud-glucose-agar.

We also analyzed the produced amounts of Stl, since phenylspirodrimanes (e.g., stachybotrylactam, Stl) are constantly discussed as an immunosuppressive agent during the sicknesses caused by *S. chartarum* genotype S in humans and animals [27,37,47]. Jagels et al. [27] detected higher amounts of phenylspirodrimanes than MCT on cellulose-containing building materials. This correlates to the current finding that the highest concentrations of Stl were detectable on CEL (5093.3 ± 429.0–7442.6 ± 3336.9 ng/g) (Figure 3). In contrast, growth on PDA resulted in large amounts of MCT, but only comparatively small amounts of Stl. Low amounts were also detected on GYP and SAB (645.2 ± 119.4 ng/g–2825.4 ± 482.5 ng/g).

In conclusion, the data of this study demonstrate that different nutrition media have a massive impact on the production and detection of MCT. This has to be taken into account if analyzing *Stachybotrys* strains for their production of MCT and defining their toxic potential. CEL seems to be the best-suited medium for toxicological studies for *Stachybotrys* spp. CEL improves the production of macrocyclic trichothecenes and phenylspirodrimanes alike. PDA is also feasible for MCT studies or MCT standard production but not so well suited for studies on stachybotrylactam.

The strains showed significant differences in toxin production, which should be further investigated. One drawback of this study is the small number of strains used. This was due to the highly complex LC-MS/MS extraction and measurement method. Every strain was analyzed on five different media as biological triplicate out of one working culture, with as little bias due to culturing as possible. Further studies should be done to better define factors influencing the production of MCT, e.g., on salts, vitamins, and amino acids, and to determine whether polysaccharides or nitrogen are limiting or repressing factors for MCT production.

## Figures and Tables

**Table 1:** Nutrition media and their ingredients.

Nutrition Media	Ingredients
MEA *	Malt-extract-agar (Merck, Darmstadt, Germany) 48 g malt-extract-agar Malted barley (reduced sugars, mainly disaccharides) 1 L aqua dest. (Evoqua Water Technologies Ultra Clear ™ TP UV UF TM, Günzburg, Germany)
PDA *	Potato-dextrose-agar (Merck, Darmstadt, Germany) 39 g potato-dextrose-agar 4 g potato infusion (200 g potatoes); 20 g D(+)glucose; 15 g agar-agar 1 L aqua dest. (Evoqua Water Technologies Ultra Clear ™, Günzburg, Germany)
GYP	Glucose-yeast-peptone-agar: 20 g glucose (Merck, Darmstadt, Germany) 5 g Yyeast-extract (OXOID, Wesel, Germany) 10 g peptone (Merck, Darmstadt, Germany) 20 g technical Agar no. 2 (OXOID, Wesel, Germany) 1 L aqua dest. (Evoqua Water Technologies Ultra Clear ™, Günzburg, Germany)
CEL	ATCC medium Cellulose Agar: 0.5 g ammonium sulfate (Merck, Darmstadt, Germany) 0.5 g L-asparagine (Merck, Darmstadt, Germany) 1 g potassium hydrogen phosphate (Merck, Darmstadt, Germany) 0.5 g potassium chloride (Merck, Darmstadt, Germany) 0.5 g yeast-extract (Merck, Darmstadt, Germany) 20 g technical Agar no. 2 (OXOID, Wesel, Germany) 10 g cellulose (Sigma Life Science, Darmstadt, Germany) 1 L aqua dest. (Evoqua Water Technologies Ultra Clear ™, Günzburg, Germany)
SAB *	Sabouraud-4% glucose-agar (Merck, Darmstadt, Germany) 65 g sabouraud-4% glucose-agar 5 g peptone (casein); 5 g peptone (meat); 40 g D(+)glucose; 15 g agar-agar 1 L aqua dest. (Evoqua Water Technologies Ultra Clear ™, Günzburg, Germany)

MEA: Malt-extract-agar; PDA: Potato-dextrose-agar; GYP: Glucose-yeast-peptone-agar; CEL: Cellulose-agar; SAB: Sabouraud-glucose-agar; *: according to the manufacturer.

**Table 2:** Limit of detection and limit of quantification.

Target	MEA [ng/g]		PDA [ng/g]		GYP [ng/g]		CEL [ng/g]		SAB [ng/g]	
	LOD	LOQ	LOD	LOQ	LOD	LOQ	LOD	LOQ	LOD	LOQ
RA	0.1	0.4	0.1	0.3	0.2	1.0	0.2	0.4	0.1	0.2
RE *	7.8	24.7	7.4	22.4	11.8	36.6	12.7	39.6	7.6	23.5
RL-2 *	0.6	1.6	1.3	6.6	1.4	6.7	0.1	0.7	2.3	10.1
SF *	0.6	1.7	0.5	1.7	3.9	12.7	0.4	1.4	1.6	6.0
SG *	0.9	3.6	0.6	1.7	2.1	7.0	0.8	2.6	0.5	1.6
SH *	1.7	6.4	0.9	4.5	7.5	24.0	1.4	6.1	14.5	44.5
VA	0.4	1.5	0.3	0.9	0.6	2.3	0.4	1.0	0.2	1.4
VJ *	0.1	0.9	0.1	0.6	0.2	1.1	0.2	0.8	0.1	0.7
Stl.	1.8	5.8	1.5	4.4	3.9	12.9	2.5	8.0	1.7	4.8

RA: roridin A; RE: roridin E; RL-2: roridin L-2; SF: satratoxin F; SG: satratoxin G; SH; satratoxin H; VJ: verrucarin J, VA: verrucarin A; Stl.: stachybotrylactam; MEA: Malt-extract-Agar; PDA: Potato-Dextrose-Agar; GYP: Glucose-yeast-peptone-agar; CEL: Cellulose-agar; SAB: Sabouraud-glucose-agar; LOD: limit of detection; LOQ: limit of quantification; *: Equivalents of roridin A.

## Supplementary Materials

The following are available online at https://www.mdpi.com/2309-608X/6/3/159/s1, Figure S1: Macroscopic appearance of *Stachybotrys chartarum* genotype S (ATCC34916) on different nutrition media; Table S1: Recovery rates for macrocyclic trichothecenes and stachybotrylactam on different nutrition media.
