# Supplementary material for: Toxin Production by Stachybotrys chartarum Genotype S on Different Culture Media
Source: J Fungi (Basel). 2020 Sep 2;6(3):159. doi: 10.3390/jof6030159 (PMC7559122; doi:10.3390/jof6030159)
Supplement: Supplementary file 1 [file jof-06-00159-s001.zip › Figures/20200705 - Figure S1 - SU.docx]

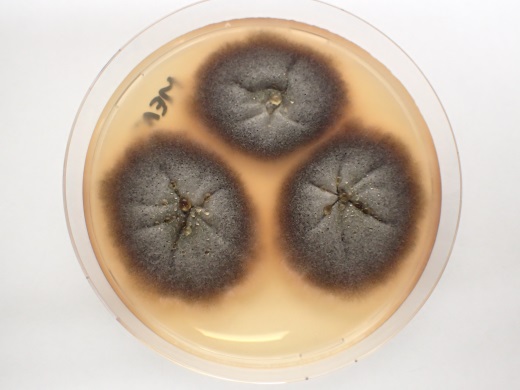

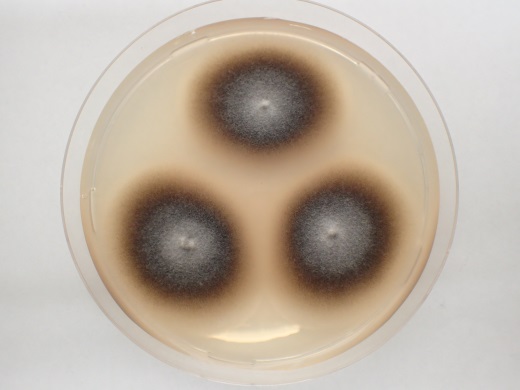


Potato-dextrose-agar (PDA)

Malt-extract-agar (MEA)

Glucose-yeast-peptone (GYP)

Cellulose-agar (CEL)


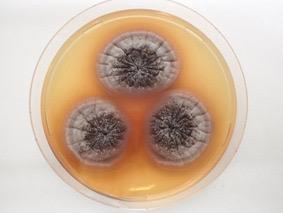

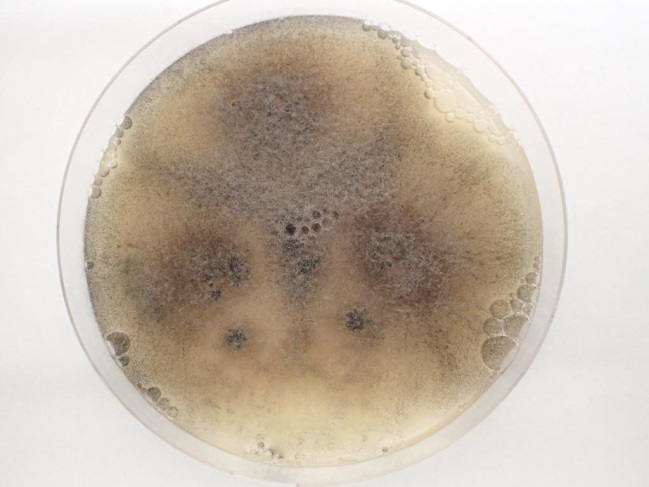

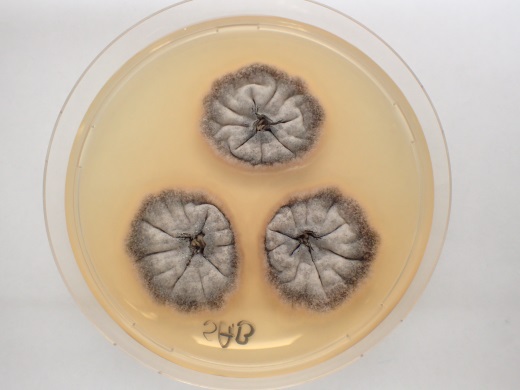


Sabouraud-dextrose-agar (SAB)

Figure S1: Macroscopic appearance of *Stachybotrys chartarum* genotype S (ATCC34916) on different nutrition media
