# Supplementary material for: Toxin Production by Stachybotrys chartarum Genotype S on Different Culture Media
Source: J Fungi (Basel). 2020 Sep 2;6(3):159. doi: 10.3390/jof6030159 (PMC7559122; doi:10.3390/jof6030159)
Supplement: Supplementary file 1 [file jof-06-00159-s001.zip › Tables/20200705 - Table S1 - SU.docx]

Table S1: Recovery rates for macrocyclic trichothecenes and stachybotrylactam on different nutrition media

| Target | MEA | | PDA | | GYP | | CEL | | SAB | |
| --- | --- | --- | --- | --- | --- | --- | --- | --- | --- | --- |
|  | Rr-Spike level (%) | | Rr-Spike level (%) | | Rr-Spike level (%) | | Rr-Spike level (%) | | Rr-Spike level (%) | |
|  | 40 ng/g | 100 ng/g | 40 ng/g | 100 ng/g | 40 ng/g | 100 ng/g | 40 ng/g | 100 ng/g | 40 ng/g | 100 ng/g |
| RA | 92±1.1 | 89±2.6 | 96±1.9 | 93±2.1 | 99±8.4 | 100±1.1 | 97±1.7 | 95±4.3 | 94±2.8 | 89±1.5 |
| RE | 78±2.8 | 69±4.6 | 101±3.5 | 88±4.8 | 102±3.9 | 95±6.4 | 92±4.2 | 84±3.6 | 120±8.4 | 114±11.3 |
| RL-2 | 136±6.7 | 92±1.5 | 130±10.0 | 87±0.8 | 99±0.3 | 86±7.9 | 116±7.4 | 89±3.5 | 120±4.0 | 88±1.2 |
| SF | 89±1.5 | 87±3.7 | 85±3.6 | 82±1.1 | 93±3.1 | 91±6.8 | 89±0.9 | 84±0.6 | 97±1.7 | 84±4.2 |
| SG | 90±2.3 | 90±1.5 | 98±3.5 | 97±2.4 | 101±8.9 | 102±4.4 | 98±1.4 | 99±2.2 | 84±5.8 | 86±2.1 |
| SH | 101±5.6 | 85±7.0 | 98±1.3 | 125±5.2 | 88±1.4 | 88±9.7 | 97±5.6 | 95±4.2 | 92±3.8 | 88±2.3 |
| VJ | 57±4.7 | 58±1.1 | 57±1.3 | 49±6.9 | 63±5.6 | 67±6.0 | 59±2.3 | 61±5.4 | 60±2.0 | 62±2.4 |
| VA | 80±2.4 | 79±1.0 | 81±2.5 | 68±6.0 | 91±7.1 | 93±0.6 | 83±2.2 | 84±3.5 | 85±4.4 | 85±1.7 |
| Stl. | 93±1.1 | 88±2.2 | 97±2.5 | 94±2.4 | 101±6.5 | 98±1.0 | 98±2.4 | 94±3.7 | 103±5.3 | 98±9.6 |

RA: roridin A; RE: roridin E; RL-2: roridin L-2; SF: satratoxin F; SG: satratoxin G; SH: satratoxin H; VJ: verrucarin J, VA: verrucarin A; Stl.: stachybotrylactam; MEA: Malt-extract-agar; PDA: Potato-dextrose-agar; GYP: Glucose-yeast-peptone-agar; CEL: Cellulose-agar; SAB: Sabouraud-glucose-agar; Rr: Recovery rate
